# Virginia Medical Monthly

**Published:** 1878-12

**Authors:** 


*Virginia Medical Monthly.*

No exchange is more welcomed than this really worthy monthly. Dr. Edwards is making it equal to any of its more pretentious Northern rivals. The dentist who wishes to enlarge the sphere of his reading (and who in this day does not wish to go outside of the strict routine of dentistry;) will find this worth the subscription. The present (Nov.) No., has an article from Dr. Cutter, of Boston, on *Rhizopods,* (*Asthmatos Ciliaris*) the drift of which is to show that if all catarrhs and influenzas are caused not by root-footed animals, at least that some are; and the evidence seems strong. H.

